# Guidelines and recommendations on yeast cell death nomenclature

**DOI:** 10.15698/mic2018.01.607

**Published:** 2018-01-01

**Authors:** Didac Carmona-Gutierrez, Maria Anna Bauer, Andreas Zimmermann, Andrés Aguilera, Nicanor Austriaco, Kathryn Ayscough, Rena Balzan, Shoshana Bar-Nun, Antonio Barrientos, Peter Belenky, Marc Blondel, Ralf J. Braun, Michael Breitenbach, William C. Burhans, Sabrina Büttner, Duccio Cavalieri, Michael Chang, Katrina F. Cooper, Manuela Côrte-Real, Vítor Costa, Christophe Cullin, Ian Dawes, Jörn Dengjel, Martin B. Dickman, Tobias Eisenberg, Birthe Fahrenkrog, Nicolas Fasel, Kai-Uwe Fröhlich, Ali Gargouri, Sergio Giannattasio, Paola Goffrini, Campbell W. Gourlay, Chris M. Grant, Michael T. Greenwood, Nicoletta Guaragnella, Thomas Heger, Jürgen Heinisch, Eva Herker, Johannes M. Herrmann, Sebastian Hofer, Antonio Jiménez-Ruiz, Helmut Jungwirth, Katharina Kainz, Dimitrios P. Kontoyiannis, Paula Ludovico, Stéphen Manon, Enzo Martegani, Cristina Mazzoni, Lynn A. Megeney, Chris Meisinger, Jens Nielsen, Thomas Nyström, Heinz D. Osiewacz, Tiago F. Outeiro, Hay-Oak Park, Tobias Pendl, Dina Petranovic, Stephane Picot, Peter Polčic, Ted Powers, Mark Ramsdale, Mark Rinnerthaler, Patrick Rockenfeller, Christoph Ruckenstuhl, Raffael Schaffrath, Maria Segovia, Fedor F. Severin, Amir Sharon, Stephan J. Sigrist, Cornelia Sommer-Ruck, Maria João Sousa, Johan M. Thevelein, Karin Thevissen, Vladimir Titorenko, Michel B. Toledano, Mick Tuite, F.-Nora Vögtle, Benedikt Westermann, Joris Winderickx, Silke Wissing, Stefan Wölfl, Zhaojie J. Zhang, Richard Y. Zhao, Bing Zhou, Lorenzo Galluzzi, Guido Kroemer, Frank Madeo

**Affiliations:** 1 Institute of Molecular Biosciences, NAWI Graz, University of Graz, Graz, Austria.; 2 Centro Andaluz de Biología, Molecular y Medicina Regenerativa-CABIMER, Universidad de Sevilla, Sevilla, Spain.; 3 Department of Biology, Providence College, Providence, USA.; 4 Department of Biomedical Science, University of Sheffield, Sheffield, United Kingdom.; 5 Department of Physiology and Biochemistry, University of Malta, Msida, Malta.; 6 Department of Biochemistry and Molecular Biology, George S. Wise Faculty of Life Sciences, Tel Aviv University, Tel Aviv, Israel.; 7 Department of Biochemistry and Molecular Biology, University of Miami Miller School of Medicine, Miami, USA.; 8 Department of Neurology, University of Miami Miller School of Medi-cine, Miami, USA.; 9 Department of Molecular Microbiology and Immunology, Brown University, Providence, USA.; 10 Institut National de la Santé et de la Recherche Médicale UMR1078, Université de Bretagne Occidentale, Etablissement Français du Sang Bretagne, CHRU Brest, Hôpital Morvan, Laboratoire de Génétique Moléculaire, Brest, France.; 11 Institute of Cell Biology, University of Bayreuth, Bayreuth, Germany.; 12 Department of Cell Biology and Physiology, Division of Genetics, University of Salzburg, Salzburg, Austria.; 13 Department of Molecular and Cellular Biology, Roswell Park Cancer Institute, Buffalo, NY, USA.; 14 Department of Molecular Biosciences, The Wenner-Gren Institute, Stockholm University, Stockholm, Sweden.; 15 Department of Biology, University of Florence, Firenze, Italy.; 16 European Research Institute for the Biology of Ageing, University of Groningen, University Medical Center Groningen, Groningen, The Netherlands.; 17 Dept. Molecular Biology, Graduate School of Biomedical Sciences, Rowan University, Stratford, USA.; 18 Center of Molecular and Environmental Biology, Department of Biology, University of Minho, Braga, Portugal.; 19 Instituto de Investigação e Inovação em Saúde, Universidade do Porto, Porto, Portugal.; 20 Instituto de Biologia Molecular e Celular, Universidade do Porto, Porto, Portugal.; 21 Departamento de Biologia Molecular, Instituto de Ciências Biomédicas Abel Salazar, Universidade do Porto, Porto, Portugal.; 22 CNRS, University of Bordeaux CBMN (UMR 5248), Pessac, France.; 23 School of Biotechnology and Biomolecular Sciences, University of New South Wales, Sydney, Australia.; 24 Department of Biology, University of Fribourg, Fribourg, Switzerland.; 25 Institute for Plant Genomics and Biotechnology, Texas A&M University, Texas, USA.; 26 BioTechMed Graz, Graz, Austria.; 27 Laboratory Biology of the Nucleus, Institute for Molecular Biology and Medicine, Université Libre de Bruxelles, Charleroi, Belgium.; 28 Department of Biochemistry, University of Lausanne, Lausanne, Switzerland.; 29 Laboratoire de Biotechnologie Moléculaire des Eucaryotes, Center de Biotechnologie de Sfax, Sfax, Tunisia.; 30 Institute of Biomembranes, Bioenergetics and Molecular Biotechnologies, National Research Council, Bari, Italy.; 31 Department of Chemistry, Life Sciences and Environmental Sustainability, University of Parma, Parma, Italy.; 32 Kent Fungal Group, School of Biosciences, University of Kent, Canterbury, United Kingdom.; 33 Faculty of Biology, Medicine and Health, The University of Manchester, Manchester, United Kingdom.; 34 Department of Chemistry and Chemical Engineering, Royal Military College, Kingston, Ontario, Canada.; 35 Zürich, Switzerland.; 36 Department of Biology and Chemistry, University of Osnabrück, Osnabrück, Germany.; 37 Heinrich Pette Institute, Leibniz Institute for Experimental Virology, Hamburg, Germany.; 38 Cell Biology, University of Kaiserslautern, Kaiserslautern, Germany.; 39 Department of Systems Biology, University of Alcalá, Alcalá de Henares, Spain.; 40 Division of Internal Medicine, The University of Texas MD Anderson Cancer Center, Houston, Texas, USA.; 41 Life and Health Sciences Research Institute (ICVS), School of Health Sciences, University of Minho, Minho, Portugal.; 42 ICVS/3B’s - PT Government Associate Laboratory, Braga/Guimarães, Portugal.; 43 Institut de Biochimie et de Génétique Cellulaires, UMR5095, CNRS & Université de Bordeaux, Bordeaux, France.; 44 Department of Biotechnolgy and Biosciences, University of Milano-Bicocca, Milano, Italy.; 45 Instituto Pasteur-Fondazione Cenci Bolognetti - Department of Biology and Biotechnology "C. Darwin", La Sapienza University of Rome, Rome, Italy.; 46 Sprott Center for Stem Cell Research, Ottawa Hospital Research Institute, The Ottawa Hospital, Ottawa, Canada.; 47 Department of Cellular and Molecular Medicine, University of Ottawa, Ottawa, Canada.; 48 Department of Medicine, Division of Cardiology, University of Ottawa, Ottawa, Canada.; 49 Institute of Biochemistry and Molecular Biology, ZBMZ, Faculty of Medicine, University of Freiburg, Freiburg, Germany.; 50 Department of Biology and Biological Engineering, Chalmers University of Technology, Gothenburg, Sweden.; 51 Novo Nordisk Foundation Center for Biosustainability, Chalmers University of Technology, Gothenburg, Sweden.; 52 Novo Nordisk Foundation Center for Biosustainability, Technical University of Denmark, DK2800 Lyngby, Denmark.; 53 Institute for Biomedicine, Sahlgrenska Academy, University of Gothenburg, Gothenburg, Sweden.; 54 Institute for Molecular Biosciences, Goethe University, Frankfurt am Main, Germany.; 55 Department of Experimental Neurodegeneration, Center for Nanoscale Microscopy and Molecular Physiology of the Brain, Center for Biostructural Imaging of Neurodegeneration, University Medical Center Göttingen, Göttingen, Germany.; 56 Max Planck Institute for Experimental Medicine, Göttingen, Germany.; 57 Institute of Neuroscience, The Medical School, Newcastle University, Framlington Place, Newcastle Upon Tyne, NE2 4HH, United Kingdom.; 58 CEDOC, Chronic Diseases Research Centre, NOVA Medical School, Faculdade de Ciências Médicas, Universidade NOVA de Lisboa, Lisboa, Portugal.; 59 Department of Molecular Genetics, The Ohio State University, Columbus, OH, USA.; 60 Malaria Research Unit, SMITh, ICBMS, UMR 5246 CNRS-INSA-CPE-University Lyon, Lyon, France.; 61 Institut of Parasitology and Medical Mycology, Hospices Civils de Lyon, Lyon, France.; 62 Department of Biochemistry, Faculty of Natural Sciences, Comenius University in Bratislava, Bratislava, Slovak Republic.; 63 Department of Molecular and Cellular Biology, College of Biological Sciences, UC Davis, Davis, California, USA.; 64 Biosciences, University of Exeter, Exeter, United Kingdom.; 65 Department of Cell Biology and Physiology, Division of Genetics, University of Salzburg, Salzburg, Austria.; 66 Institute of Biology, Division of Microbiology, University of Kassel, Kassel, Germany.; 67 Department of Ecology, Faculty of Sciences, University of Malaga, Malaga, Spain.; 68 A.N. Belozersky Institute of physico-chemical biology, Moscow State University, Moscow, Russia.; 69 School of Plant Sciences and Food Security, Faculty of Life Sciences, Tel Aviv University, Tel Aviv, Israel.; 70 Institute for Biology/Genetics, Freie Universität Berlin, Berlin, Germany.; 71 Laboratory of Molecular Cell Biology, Institute of Botany and Microbiology, KU Leuven, Leuven, Belgium.; 72 Center for Microbiology, VIB, Leuven-Heverlee, Belgium.; 73 Centre of Microbial and Plant Genetics, KU Leuven, Leuven, Belgium.; 74 Biology Department, Concordia University, Montreal, Canada.; 75 Institute for Integrative Biology of the Cell (I2BC), SBIGEM, CEA-Saclay, Université Paris-Saclay, Gif-sur-Yvette, France.; 76 Department of Biology, Functional Biology, KU Leuven, Leuven-Heverlee, Belgium.; 77 Cevec Pharmaceuticals, Cologne, Germany.; 78 Institute of Pharmacy and Molecu-lar Biotechnology, Heidelberg University, Heidelberg, Germany.; 79 Department of Zoology and Physiology, University of Wyoming, Laramie, USA.; 80 Department of Pathology, University of Maryland School of Medicine, Baltimore, USA.; 81 School of Life Sciences, Tsinghua University, Beijing, China.; 82 Department of Radiation Oncology, Weill Cornell Medical College, New York, NY, USA.; 83 Sandra and Edward Meyer Cancer Center, New York, NY, USA.; 84 Université Paris Descartes/Paris V, Paris, France.; 85 Equipe 11 Labellisée Ligue Contre le Cancer, Centre de Recherche des Cordeliers, Paris, France.; 86 Cell Biology and Metabolomics Platforms, Gustave Roussy Comprehensive Cancer Center, Villejuif, France.; 87 INSERM, U1138, Paris, France.; 88 Université Pierre et Marie Curie/Paris VI, Paris, France.; 89 Pôle de Biologie, Hôpital Européen Georges Pompidou, Paris, France.; 90 Institute, Department of Women’s and Children’s Health, Karolinska University Hospital, Stockholm, Sweden.

**Keywords:** accidental cell death, apoptosis, autophagic cell death, autophagy, caspases, mitochondrial membrane permeabilization, mitotic catastrophe, model organism, necrosis, reactive oxygen species, regulated cell death, Saccharomyces cerevisiae

## Abstract

Elucidating the biology of yeast in its full complexity has major implications for science, medicine and industry. One of the most critical processes determining yeast life and physiology is cellular demise. However, the investigation of yeast cell death is a relatively young field, and a widely accepted set of concepts and terms is still missing. Here, we propose unified criteria for the definition of accidental, regulated, and programmed forms of cell death in yeast based on a series of morphological and biochemical criteria. Specifically, we provide consensus guidelines on the differential definition of terms including apoptosis, regulated necrosis, and autophagic cell death, as we refer to additional cell death routines that are relevant for the biology of (at least some species of) yeast. As this area of investigation advances rapidly, changes and extensions to this set of recommendations will be implemented in the years to come. Nonetheless, we strongly encourage the authors, reviewers and editors of scientific articles to adopt these collective standards in order to establish an accurate framework for yeast cell death research and, ultimately, to accelerate the progress of this vibrant field of research.

## INTRODUCTION

Yeast, a fungus that predominantly lives as a unicellular organism, has had an extraordinary influence on humanity throughout millennia, from its usage for baking and brewing to the potential of some species to cause life-threatening human diseases. The cultural, industrial, biotechnological, and medical impact of this organism remains unparalleled. The use of yeast fermentation to produce alcoholic beverages and to leaven bread coincided with the rise of ancient civilizations and has persisted until our days. Importantly, the continued development of yeast strains as vehicles for the development of new technology, for example in bioethanol, drug, and enzyme production, as well as the implementation of unconventional yeast species in industrial processes, highlights the ever increasing importance of yeast now and in the future 1,2. This is exemplified by the fact that the global market for yeast products is in the multibillion dollar range and is expected to grow further 3. Beyond the mentioned applications, yeast has a direct impact on human health and disease. Many fungi, including some yeasts, can exist as commensals, i.e., they are part of our natural microbiota, forming the mycobiome 4. In fact, it is being increasingly recognized that fungi are a major determinant in establishing commensal microbial communities and are thus vital for healthy individuals 5. However, under certain circumstances, e.g., compromised immunity, commensal fungi may become opportunistic pathogens and as such are a potential cause for infectious diseases 6. These include superficial infections of the skin and nails (especially by dermatophytes) that affect billions worldwide, biofilm colonisations of mucosal surfaces and more serious invasive infections, which can have very high mortality rates and are estimated to lead to 1.5 million deaths per year 7. A significant number of these deaths arise from infections caused by the yeasts *Candida albicans*,* Candida glabrata *and* Cryptococcus neoformans* in immunocompromised individuals. This socioeconomic burden is further amplified by the unprecedented rise in fungal diseases that are affecting plants and animals 8. These examples highlight the importance of a full understanding of fungal biology, and the study of yeast cell biological processes has been crucial in this respect.

Yeasts have served as a successful research tool for the last century, *Saccharomyces cerevisiae* (the budding yeast) being one of the most thoroughly studied eukaryotes at the cellular and molecular levels. Indeed, yeast continues to be one of the preferred model organisms to explore eukaryotic cell biology, both due to its technical advantages in devising/sophisticating molecular tool kits to study cellular biology, and to a high degree of functional conservation 9. Also, yeast offers rapid growth and inexpensive accessibility paired with a high amenability to biochemical and genetic manipulation. This enables the establishment of various experimental setups, ranging from single experiments to high-throughput, genome-scale, unbiased screenings in a short time frame. Notably, many insights obtained in yeast have proven to be transferable to higher eukaryotes. Indeed, over the past decades, yeast studies have unveiled individual gene functions as well as gene and protein interactions, and have instrumentally contributed to the understanding of fundamental cellular processes such as eukaryotic cell cycle control 10,11,12,13,14,15, autophagy 16,17,18,19, mitochondrial function 20,21, including mitochondrial import 22,23,24,25, protein degradation 26, vesicle fusion 27,28, genetic instability 29,30, epigenetic control 31,32, metabolic regulation 33,34,35, or cellular nutrient sensing 36.

In addition, studies on yeast have shed light on human diseases, providing a cellular platform to examine, for instance, prion biology, virus-host interactions, metabolic diseases, neurodegenerative disorders, cancer, or aging 37,38,39,40,41,42,43,44,45,46,47,48,49,50,51,52,53,54,55,56,57,58,59,60,61. Among the pathophysiologically relevant pathways that can readily be explored in yeast are those governing cellular demise. Indeed, cell death regulation is structurally and functionally conserved in yeast 21,62,63,64,65,66, and yeast has even served to uncover and establish factors and pathways involved in apoptosis and other controlled cell death subroutines, which have later been corroborated in metazoan or other multicellular systems, e.g., the AAA-ATPase Cdc48/VCP 63,67, the BAX inhibitor-1 68, the implication of metacaspases as cell death regulators 69,70,71, the role of cathepsin D in non-autophagic mitochondrial degradation 72,73, or the lethal impact of ER-Golgi transport blockage as one of the mechanisms explaining the demise of dopaminergic neurons during Parkinson’s disease 74. To sum up, on the one hand, cell death represents a key process that can be feasibly modeled in yeast. On the other hand, the understanding of yeast cell death and its putative modulation may improve industrial and biotechnological applications, provide insights into mycobiome dynamics, and help develop the fight against fungal and other diseases.

In multicellular organisms, the controlled suicide of single cells is crucial for development and homeostasis, providing a system that eliminates superfluous cells. The presence of such a mechanism also allows for the removal of damaged cells that might compromise organismal fitness. In a single-celled organism like yeast, this paradigm does not seem to apply at first sight, since - in this case - cellular suicide entails the death of the whole organism. However, in a way, a population of yeast cells *de facto* behave as a multicellular entity of communicating individuals rather than a group of isolated cells that do not interact with each other. In fact, a given yeast population originates from a single clone, and the ultimate biological goal of that population is the survival of the genetic information representing that very clone. Thus, under certain circumstances, the death of unfit or damaged yeast cells promotes the survival of the population as a whole. A number of physiological scenarios have been described that corroborate this teleological explanation for a cellular suicide program in yeast, including antagonistic interactions between yeasts, aging, mating, or colony formation 54,61,75,76,77,78,79,80,81,82,83,84,85. Of note, also other unicellular organisms, including bacteria and protozoan parasites, incorporate regulatory processes that are at least partly reminiscent of higher eukaryotic cell death programs 86,87,88,89,90,91.

**Figure 1:**
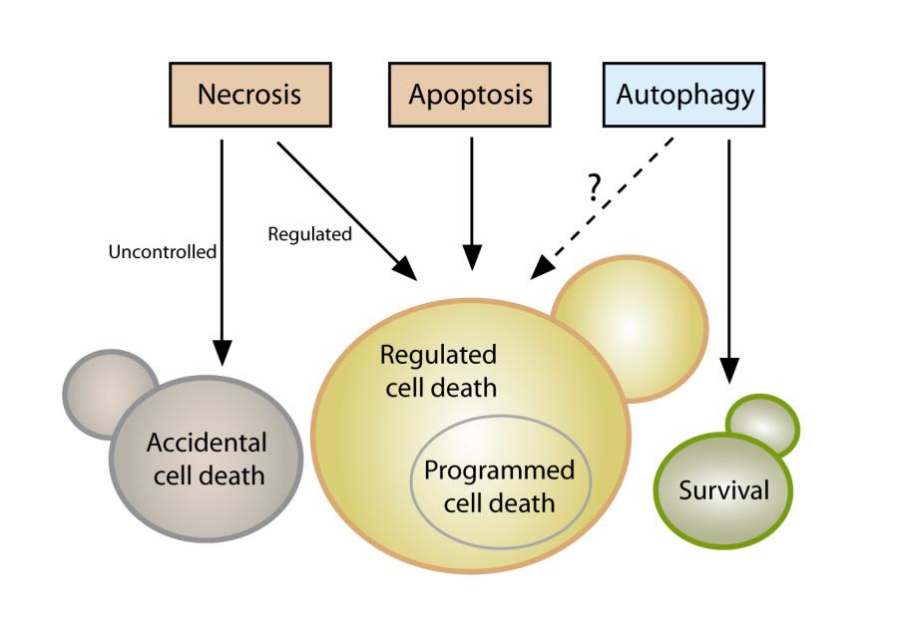
FIGURE 1: Yeast cell death. Yeast cells can die either upon exposure to very harsh microenvironmental conditions via accidental cell death (ACD) or in the context of a failing response to mild stress via regulated cell death (RCD). While ACD invariably manifests with a necrotic morphotype (disintegration of cell structure, plasma membrane rupture), RCD can exhibit a spectrum of morphologies and can result from multiple signaling pathways, including regulated necrosis or apoptosis. Programmed cell death (PCD), which occurs in strictly physiological scenarios (e.g., development), represents a specific type of RCD. The possible role of autophagy as a cell death pathway in yeast remains elusive, while its cytoprotective function is well established.

Even though it is now clear that yeast can indeed undergo cellular suicide, the corresponding terminology to describe this multifaceted process remains heterogenous and potentially misleading. Thus, we believe that there is timely need for a more precise and consistent nomenclature that clearly defines the concept of “yeast cell death”, considering morphological, enzymological, and functional aspects. Such standardization seems of importance, given that the field of yeast cell death is continuously expanding with significant progress being made at the phenotypical and mechanistic levels, including the finding that, akin to higher eukaryotes, yeast can also engage in distinct cell death modalities (**Figure 1**). In this paper we thus attempt for the first time to formulate a series of recommendations and caveats with respect to cell death-related results obtained in yeast. To this aim, we have followed the directions of the Nomenclature Committee on Cell Death (NCCD) 92,93,94,95 and adapted them to the particularities of *Saccharomyces cerevisiae*, which we think can be extended to other yeast species and to multicellular filamentous fungi. Our goal is to frame a uniform set of guidelines that facilitate the communication among yeast cell death researchers, ultimately supporting and accelerating scientific advance (**Box 1**). In that respect, the nomenclature presented herein will likely need to be revised and updated as the field of yeast cell death moves forward and even more precise definitions are required.

**Box 1:**
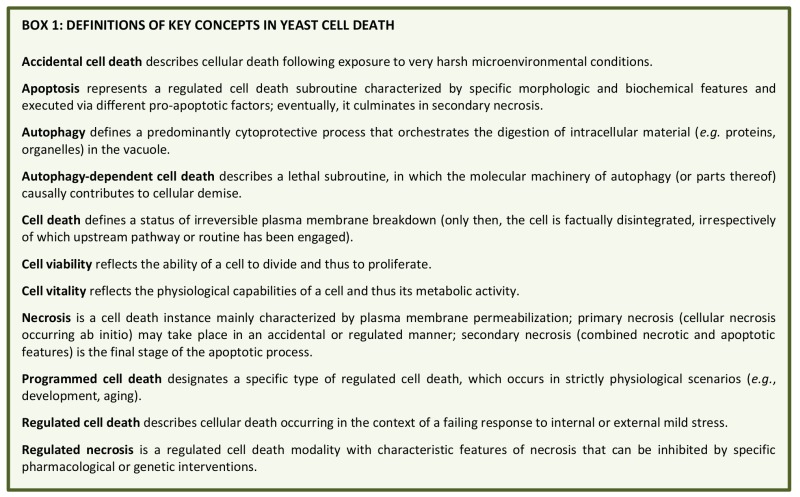


## YEAST CELL DEATH AND SURVIVAL

A crucial issue that demands a clear definition is the question of cell death itself. When is a cell dead? According to the NCCD guidelines this is only the case upon irreversible plasma membrane breakdown or complete cellular fragmentation, because only then the cell is factually disintegrated, irrespectively of which upstream pathway or routine has been engaged 93. In fact, no earlier marker can be defined that reliably determines death in all settings. Thereby, this lethal irreversibility might start with the collapse of the electrochemical membrane potential across the plasma membrane through formation of a leak. In yeast, the most common method to monitor cell membrane integrity *in vivo* is to use propidium iodide (PI). PI is a fluorescent nucleic acid intercalator that can only enter cells with a ruptured cell membrane, and can be routinely employed in both low and high throughput formats 96,97,98. Along similar lines, colorimetric dyes like trypan blue may be used, but are less common 99,100,101. Further potential alternatives exist (e.g., 7-aminoactinomycin D), but will need to be thoroughly tested with respect to their suitability for yeast cell death applications in future studies. As mentioned, assessing cell membrane disintegrity is the only technique to quantify actual cell death and must be performed irrespectively of the lethal setting being analyzed. This is imperative, since lethal signaling does not imply that the final stage (cell death) is reached or even that it will be reached at a later stage (see below). In fact, specific subpopulations engaged in lethal pathways that maintain plasma membrane integrity (e.g., early apoptotic cells, see below) are by definition not (yet) dead. In that respect, timecourse experiments are important to monitor both the lethal subroutine-specific phenotypes and the actual occurrence of cell death over time. Of note, indications exist that upon specific stress insults, a small subpopulation of yeast retains the ability to repair cell membrane damage even after having stained positive for PI 102. Given the lack of other comparably well established dyes in this context and the large body of data supporting PI staining as a valid method to quantify loss of survival, we conclude that determining PI positivity is - at this point - the best technique to quantitatively approach yeast cell death. Still, for the sake of accuracy and waiting for further evidence supporting the above-mentioned indications, we suggest expressing a corresponding quantification as "% PI-positivity" or "% cell death ( PI positive )" instead of "% death" or "% survival" upon using this method. In the long term, the development and establishment of alternative dyes should be explored in order to validate data obtained with PI. A number of approaches allow to experimentally assess (i) cell viability, which reflects the ability of a cell to divide, and (ii) cell vitality, defined as the physiological capabilities of a cell 100. Nonetheless, an impaired/compromised (i) proliferation or (ii) metabolic capacity does not necessarily result in cellular demise. Thus, these techniques alone cannot be used to demonstrate cell death. Still, they are very useful to complement and corroborate data obtained with PI or alternative dyes.

Assessing clonogenicity with plating assays is the most commonly used method to quantify cell viability 62,103. Here, a defined number of cells from a given culture are plated on rich medium agar plates that are further incubated to allow colony formation. The ratio between the resulting colony-forming units (CFUs) and the originally plated number of cells reflects the viability state in the culture. Theoretically, however, it is possible that under specific conditions (of genetic nature, for instance), colony formation may be blocked in cells that *per se* are still alive (a condition usually refered to as senescence). Additional caveats include the possibility that live cells at the point of plating might die before forming a colony and/or that the plating procedure itself might drive (a fraction of) cells into death, which would be indistinguishable from cell senescence. Nonetheless, the literature suggests clonogenic capacity as a very good correlate to cell death in a plethora of different settings 69,96,104,105 and thus represents a valid approximation to quantify survival in yeast populations. Of note, clonogenicity can also be measured by monitoring CFU formation at the microcolony level (time-lapse photomicroscopy) 106,107. Even though cell and colony counting can be automated, clonogenicity assays are rather time-consuming and used for low- to medium-throughput analyses.

A further technique to assess yeast viability follows the growth rate of a given culture, which may decrease as a consequence of increased cell death. For this purpose, an aliquot is inoculated into fresh liquid medium and the growth is monitored, for instance, via photometric measurement of optical densities over a specific period of time 108,109. Optionally, spot dilution assays can be performed, where cultures are spotted in serial dilutions on agar plates 110. Here, the growth ability is compared between cultures at the various dilution steps in a semi-quantitative manner, although automated readout of microcolonies can be used to yield a quantitative result 111. Monitoring growth can be scaled up and performed either manually or using robotics support, which makes it an attractive technique, especially for screen-based analyses. As with other viability assays, an important disadvantage is that a decreased growth rate can also result from a non-lethal event such as modulation of cell cycle progression or a reduced metabolism due to an alteration in the use of media components.

**Figure 2:**
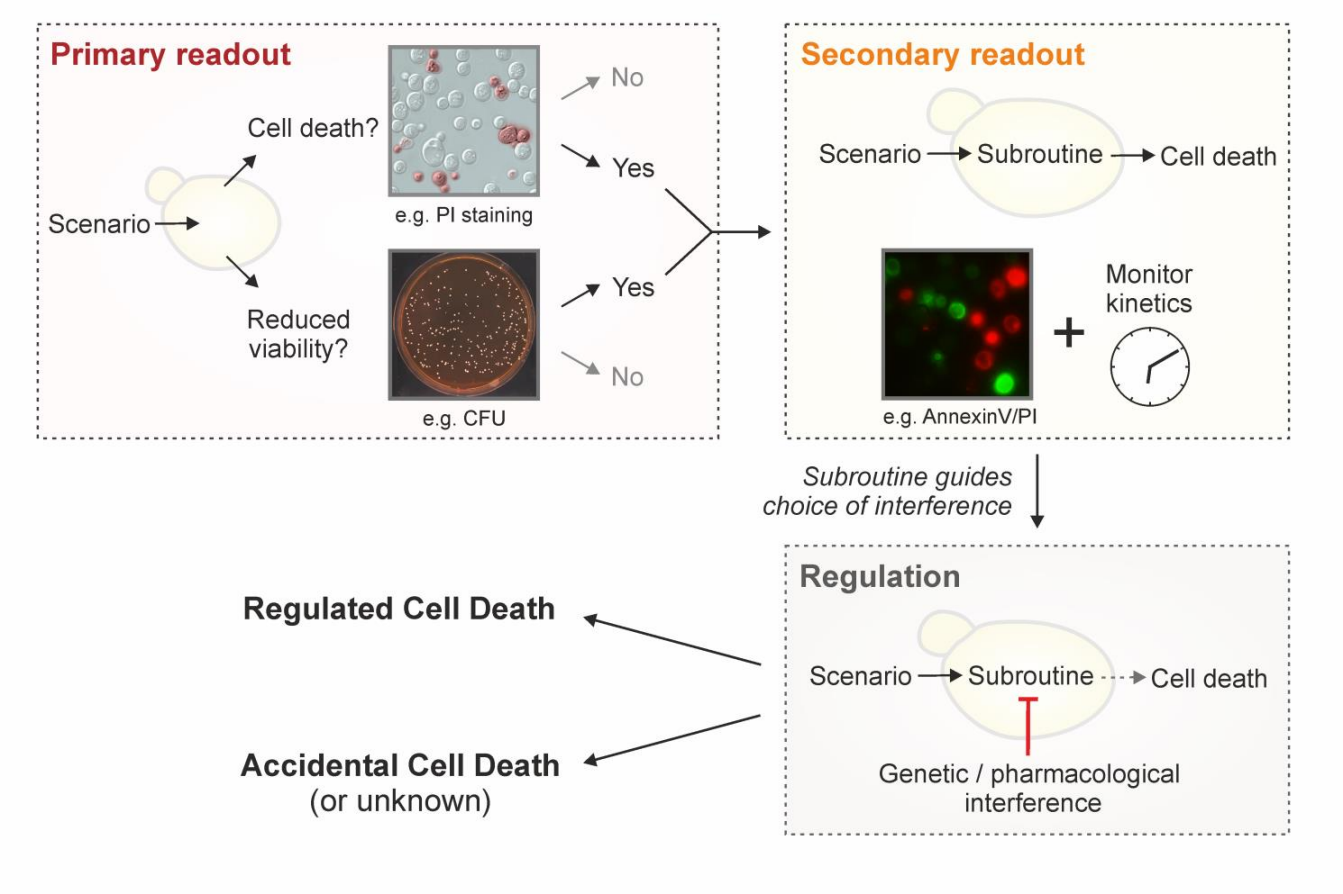
FIGURE 2: Strategy to characterize yeast cell death. To define a lethal scenario in yeast, we recommend to sequentially evaluate the following three levels. (i) The occurrence of cell death should be assessed by monitoring loss of plasma membrane integrity (e.g., by staining with exclusion dyes such as propidium iodide, PI). We suggest to complement this assessment by determining viability with clonogenic tests, knowing that, in many scenarios, clonogenic capacity correlates exceptionally well with cell survival. Other viability and vitality assays may be performed to corroborate the results obtained, but do not replace these two assays. (ii) If cell death is demonstrated, the possible RCD **subroutine(s) should be examined via morphological and biochemical observations. While necrotic and autophagic phenotypes demand a further clarification (inhibition studies) to conclude whether the observations correspond to an RCD modality (regulated necrosis, autophagic cell death), ACD (accidental necrosis), or a cell death correlate (protective autophagy), an apoptotic phenotype directly indicates RCD (via apoptosis). Irrespectively, **it is imperative to follow the scenario over time (kinetics). (iii) Regulation per se and/or assessment of the regulatory network should be tackled by means of **genetic and/or pharmacological interventions. Importantly, these interventions should inhibit or shift cell death and the observed subroutine-specific phenotypes to conclude on the involvement of an RCD modality (for regulated necrosis, autophagic cell death) and/or to provide mechanistic insight (all RCD types).**

One possibility to evaluate yeast cell vitality is to directly assess the activity of specific enzymes directly. Although this is not widely employed in yeast cell death research, it represents an avenue to assay the physiological state of a metabolic pathway within the cell 100,112,113. As pointed out below, a caveat of this approach is the possible distortion of results by residual activity in dead cells. A further option is to use vital dyes, like the two-color fluorescent probe FUN-1, which diffuses into cells, irrespectively of their viability status, and results in green fluorescence of the cytoplasm. Dead cells fluoresce green while (live) cells that have both plasma membrane integrity and metabolic capability, can further process the probe, resulting in red vacuolar fluorescence 114,115. Similarly, several tetrazolium salts are reduced into colored formazan crystals 116. Methylene blue is converted to the colorless leucomethylene blue only in metabolically active cells 117, while the red dye phloxine B is only retained in metabolically inactive cells that are unable to actively export it 100,118. Other methods aim at assessing further aspects of cellular physiology, including the cellular ATP content (e.g., based on the luciferin-luciferase reaction) 119 or mitochondrial transmembrane potential (e.g., upon staining with rhodamine 123, JC-1, TMRM/E, DiOC_6_(3)) 120,121. It should be noted that the readout of metabolic signatures has considerably improved with new generation extracellular flux analyzers, offering the possibility to simultaneously measure mitochondrial respiration and glycolysis (and thus mitochondrial function). A drawback of metabolic assays resides in the fact that cells may be able to maintain some metabolic activities until cell membrane rupture occurs, and that some rely on specific metabolic processes such as oxidative phosphorylation that are not mandatory for cell survival. Thus, such techniques may fail to detect subpopulations of dead (or alive) cells, reflecting the notion that a decrease in growth or metabolic activity (i.e*.*, viability or vitality) cannot be placed on a par with an increase in cell death. In conclusion, as mentioned above, the term cell death should be used only upon observing breakdown of the plasma membrane and thus loss of cell integrity (e.g., upon PI staining). In addition, we suggest to strengthen this observation by simultaneously assessing clonogenic capacity (**Figure 2**), since (i) it represents the best-established output to accurately monitor overall cellular viability and (ii) it empirically correlates very strongly with actual cell death markers. Importantly, both methods are easy, quick and relatively inexpensive. The use of additional dyes/stainings/assays provides valuable complementary information, but cannot be used alone to unequivocally define a cell as dead.

Yeast cell death is often accompanied by oxidative damage and thus, a widely employed method in the field is the detection of reactive oxygen species (ROS) 122. Indeed, a number of different ROS, like the superoxide anion, hydroxyl radical, and hydrogen peroxide, can accumulate upon mitochondrial disturbances, ER stress or other cellular derangements 96,122,123,124,125. ROS can generally be detected using membrane-permeable dyes that are oxidized to fluorescent products in a ROS-dependent manner. Importantly, these stains do not measure ROS as a group, but rather react with specific species. For instance, dihydroethidium (DHE) preferentially reacts with superoxides, while dihydrorhodamine 123 (DHR123) and 2,7-dichlorodihydrofluorescein diacetate (H_2_-DCF-DA) are converted by a broad range of other ROS, but only poorly by superoxides 126. Such specificities should be taken into account when measuring ROS with a particular stain, since distinct lethal triggers might result in the production of a differential ROS subset 123. Thus, we recommend to specifically indicate the ROS subtype that is being monitored instead of generally referring to ROS production. Of note, to a certain degree, DHE may also be oxidized unspecifically (independently of superoxide). In order to exploit the full potential of DHE as a superoxide-specific dye, a range of methodological possibilities (e.g., the use of optimized spectra) exist 127,128. The standardization of such refinements for DHE assays, which are a preferred tool in yeast cell death research, should be addressed in the future. While ROS measurements allow for high-throughput approaches due to their simplicity and relatively low cost, it is imperative to realize that this method does not discriminate between living and dead cells, although ROS usually precede and are often causative for cell death in yeast 125. In fact, ROS play a crucial role in intracellular signaling 129,130,131,132, functioning, for instance, as direct and indirect regulators of diverse physiologically relevant targets 133,134,135. In addition, limited ROS generation might be beneficial under certain conditions, since the resulting adaptive responses can promote stress resistance as a form of preconditioning (hormesis) 131,136,137,138,139. Thus, an increase in ROS should be regarded as a cell death-correlated phenotype only in connection with assays that directly determine increased plasma membrane disintegration and loss of clonogenicity (see above). Similarly, a decrease in ROS production by incubation with anti-oxidants might support the mechanistic involvement of ROS in the lethal process, but only when cell death is adequately monitored.

## ACCIDENTAL VERSUS REGULATED CELL DEATH

Cellular demise in yeast may occur in two mutually exclusive variants: either as an accidental event or through a regulated pathway. Accidental cell death (ACD) occurs upon exposure to severe conditions, resulting in a rapid, uncontrollable and unavoidable form of death. ACD may follow a series of extreme stimuli, including physical conditions, such as very high temperatures or pressures, severe chemical insults like strong detergents and high concentrations of acids or bases as well as mechanical challenges, for instance, vigorous shearing or ultrasonic treatment. The immediate nature of ACD, which is characterized by a virtually immediate structural breakdown of cells, allows no form of pharmacologic or genetic inhibition. Thus, this form of cell death does not constitute a direct target for modulation or prevention. However, it remains unclear whether yeast cells undergoing ACD may release endogenous, bioactive molecules to the extracellular space 75,79. If so, such molecules could interact with local cells that have survived the primary insult and ignite a response within the whole yeast population. Such a consequence of ACD may resemble the release of damage-associated molecular patterns (DAMPs) by dying human cells. DAMPs can stimulate a direct or indirect (via innate immune effectors) cytotoxic response in surrounding bystander cells that have survived ACD 140,141,142,143,144. In such a case, interfering with the effects of ACD on the rest of the population remains possible.

ACD is often equated with necrosis, which in yeast is usually identified as a cellular condition of early plasma membrane permeabilization in the absence of typical apoptotic markers and of complete disintegration of subcellular structures 103. Indeed, ACD usually exhibits morphological features of necrosis, but mounting evidence suggests that - as it is the case in human cells - a physiologically relevant, regulated type of necrosis does also exist in yeast. Thus, we recommend to avoid using the term "necrosis" to define an accidental and uncontrollable type of death, and to favor the term "ACD". We believe that this will avoid any potential misunderstandings regarding the two fundamentally dinstinct (accidental *versus* regulated) modalities of yeast cell death manifesting with a necrotic morphology (see below).

That said, many lethal stimuli result in a form of yeast cell death that - at odds with ACD - is executed by a genetically encoded, dedicated molecular machinery. In higher eukaryotes, a distinction is made between such a controlled form of cell death when it occurs (i) in the framework of a purely physiological program, e.g., during (post-) embryonic development or tissue homeostasis, or (ii) as a response to either a perturbation of intracellular or extracellular homeostasis, e.g*.*, upon exposure to mild stress or as a consequence of mutations. Cell death occurring in the former scenario is termed "programmed cell death" (PCD), while the expression "regulated cell death" (RCD) encompasses both PCD as well as all other instances of cell death that depend on a molecular machinery 145,146,147,148.

For yeast cell death, many authors have used the term PCD to interchangeably refer to all types of cellular demise that are not accidental (i.e., to all instances of RCD). However, emerging evidence is confirming that a yeast population, be it a liquid culture or a solid colony, bears a degree of complexity reminiscent of multicellular organisms that demands a revision of this terminology. For instance, during yeast gametogenesis (or sporulation), immature meiotic products as well as the mother cell itself succumb via activation of vacuolar rupture 149,150. Interestingly, the mother cell’s demise is delayed until spores have reached a threshold degree of differentiation. Thus, in this scenario, RCD occurs in the frame of a developmentally coordinated program, *de facto* representing an instance of PCD. During yeast chronological aging, the cellular community maintains homeostasis thanks to the programmed death of dysfunctional or old cells, which spares and provides nutrients to the fitter individuals 75,76. In yeast colonies, stationary-phase or slow-growing cells differentiate into specific subpopulations with unique metabolic properties and particular functions within the colony 151,152. These examples show that, indeed, yeast populations can harness cell death to control coordinated development, homeostasis and differentiation. Hence, we propose to define PCD in yeast as a specific instance of RCD that is executed in the frame of such physiologic programs. All other forms of regulated demise (e.g., cell death induction upon stress, or as a consequence of specific genetic alterations) should be referred to with the superordinate term of RCD.

Importantly, since RCD depends on a defined molecular machinery, it can be modulated with pharmacologic or genetic means. The extent of such modulation depends on the progression of the process across a hitherto poorly defined point-of-no-return. According to the NCCD, the processes preceding such point are part of cellular stress responses, while those following it belong to actual cell death signaling 93. Adopting this rationale, RCD can be accelerated or delayed (but not avoided) if the point-of-no-return has been trespassed. Instead, prior to that point, modulating stress responses or avoiding stress can prevent RCD. However, the definition of this point-of-no-return has not been established yet, implying that the exact boundary between the reversibility of a stress stimulus and the irrevocable engagement in a lethal cascade remains to be specified.

**Box 2:**
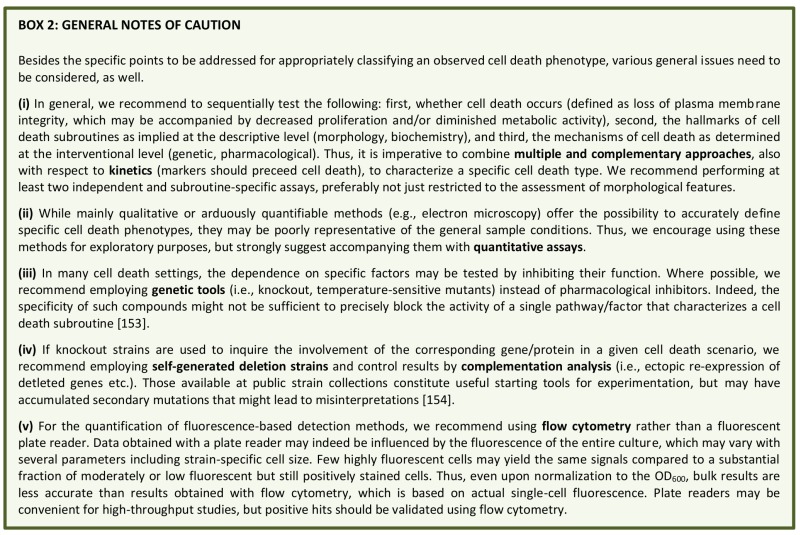


Yeast RCD may follow different subroutines (see below) that can be differentiated from each other by a series of morphological and biochemical features. To precisely characterize the lethal phenotype, we recommend (i) to first determine if cell death actually occurs (as opposed to only reduced viability/vitality), (ii) if it does, to then examine the subroutine(s) involved via morphological and biochemical observations, using at least two different detection methods 155, and (iii) finally, to corroborate the implicated mechanism(s) via genetic and pharmacological interventions (**Figure 2**). Finally, it should be noted that in cell death research, it is generally advisable to determine the kinetics of the parameters under scrutiny 156. In order to detect the differential appearance of apoptotic or necrotic characteristics, we recommend assessing such features at different time points to yield a better resolution of cell death events. Importantly, subroutine-specific markers should precede cell death. In the following sections, we will describe yeast RCD subroutines and the techniques to precisely discriminate amongst them (**Table 1**). Beyond the specificities outlined below, a number of general issues and notes of caution also need to be considered (**Box 2**).

**Table 1:** Methods commonly used for the assessment of cell death, viability and vitality as well as for the identification of different cell death subroutines in yeast. ^a^ Please note that necrotic features are common to different subpopulations and phenotypes (primary necrosis, secondary necrosis, accidental necrosis, regulated necrosis). ^b^ Please note that autophagy is often cytoprotective and that the additional experiments are necessary to to establish the occurrence of ADCD. ADCD, autophagy-dependent cell death; ALP, alkaline phosphatase; Δψ_m, _mitochondrial transmembrane potential; EM, electron microscopy; IMS, intermembrane mitochondrial space; KO, knockout; MOMP, mitochondrial outer membrane permeabilization; OD, optical density; PI, propidium iodide; ROS, reactive oxygen species; TUNEL, terminal deoxynucleotidyl transferase dUTP nick end labeling.

	**Feature**	**Method**	**Limitations**	**References**
Death	Loss of cell membrane integrity	PI, trypan blue	Does not distinguish between primary and secondary necrotic cells (unless coupled with Annexin V assay and kinetics study)	96,97,99
Viability	Colony formation	Clonogenic capacity (quantification of colony-forming units)	Slow, work-intensive, does not differentiate between cell death and senescence	69,96,104
	Growth capacity	Spot dilution	Possible non-lethal modification in cell cycle or metabolism; semi-quantitative (except for microcolony count)	110,111
	Growth capacity	OD_600_	Possible non-lethal modification in cell cycle or metabolism; concurrent growing and dying subpopulations	108,109
Vitality	Metabolic activity	Specific enzymatic activity	Possible non-lethal modification in cell cycle or metabolism	100,112,113
	Metabolic activity	Physiological parameters	Possible residual activity in factually dead cells	119,120
	Metabolic activity	General activity (e.g., FUN-1, methylene blue, phloxine B)	Possible residual activity in factually dead cells, or living cells without assayed metabolic activity	114,115,116,117,118
	Oxidative stress	ROS (e.g. DHE, DHR123, H2-DCF-DA)	ROS specificity, only correlative and non-exclusive feature	96,122,123,124,125
Apoptosis	PS externalization	AnnexinV/PI	Need of spheroplasts	63,96,104,157
	Chromatin condensation	DAPI	Not fully specific, can occur in some form of necrosis	63,125
	Chromatin condensation	EM	Slow, non compatible with quantification	63,125
	DNA fragmentation	TUNEL	Possible staining of vital processes, can occur in some forms of necrosis	63,96,104,157,158
	MOMP (release of pro-apoptotic proteins from the IMS)	Western blot, immunofluorescence microscopy, enzymatic activities of IMS proteins	Also occurs during necrosis	96,159,160,161
	Loss of Δψ_m_	Potentiometric fluorescent probe imaging or flow cytometry	Also occurs during necrosis	121,162,163,164
	Dependence on known regulators	Knockout analysis	May affect other signal cascades	62
	Dependence on de novo protein synthesis	Protein synthesis inhibitors (e.g., cycloheximide)	Interferes with the synthesis of anti-apoptotic proteins; may apply to other RCD instances	125
Necrosis^a^	Disruption of plasma membrane	EM	Slow, quantification	103,157
	Disruption of plasma membrane	Unpermeable fluorochromes (e.g. , PI)	Does not discriminate between primary and secondary necrosis	103,157
	Nucleo-cytosolic translocation of Nhp6A	Fluorescence microscopy (GFP-tag)	Does not discriminate between primary and secondary necrosis	104,157,165
	Disintegration of subcellular structures	EM	Slow, quantification	103,157
	MOMP (release of pro-apoptotic proteins from the IMS)	Western blot, immunofluorescence microscopy, enzymatic activities of IMS proteins	Also occurs during apoptosis	149
	Loss of Δψ_m_	Potentiometric fluorescent probe imaging or flow cytometry	Also occurs during apoptosis	121,149
Regulated necrosis (additional features to discriminate from accidental necrosis)	Pharmacological inhibition	Spermidine	May affect other signal cascades	157
	Genetic dependence	Inhibition by spermidine biosynthesis	May affect other signal cascades	157
	Genetic dependence	Inhibition by Pep4 propeptide	Propeptide-free Pep4 is not expressed (limited to propeptide overexpression studies)	104
Autophagy	GFP-Atg8 processing	Western blot	May occur with some degree of non-specificity	166,167
	GFP-Atg8 processing	Fluorescence microscopy	May occur with some degree of non-specificity	166,169,170
	Modified ALP activity	ALP assay	May result from other signaling pathways, results could be influenced by changes in Pho8 expression levels	171,172
	pH-change upon delivery of cellular compartments to the vacuole	Rosella fluorescent pH biosensor	May result from other signaling pathways	173
	Others	Biochemical, microscopic	-	175
ADCD (additional dependency assessment)^b^	Pharmacological inhibition (dependence on autophagic degradation)	Inhibition of vacuolar proteolysis	May affect other signal cascades	175,176
	Pharmacological inhibition (dependence on autophagic degradation)	Inhibition of autophagic process	May affect other signal cascades, insufficient specificity	175,177
	Genetic dependence (dependence on autophagic machinery)	KO of ATG genes	May affect other signal cascades	174,175
	Genetic dependence (dependence on autophagic machinery)	KO of vacuolar protolysis	May affect other signal cascades	175,176
	Genetic dependence (dependence on autophagic machinery)	Constitutive activation of TOR or RAS/PKA	May affect other signal cascades	178,179,180,181

## APOPTOSIS

Most studies on RCD in yeast have been conducted in the budding yeast *Saccharomyces cerevisiae*. This includes the first observation of an apoptotic phenotype in yeast, specifically in a strain with a point mutation in the gene coding for the cell cycle protein Cdc48 63. One of the early indications for an active cellular participation in the yeast apoptotic process was that RCD in this setting can be prevented by inhibiting *de novo* protein synthesis, e.g. by cycloheximide 125. Ever since these discoveries, a set of methods has been established, validated and refined that allows to specifically determine whether a yeast cell has engaged in an apoptotic pathway 62. These techniques are mainly based on the key morphologic and biochemical features of an apoptotic cell. We suggest employing at least two of these apoptosis-specific methods (one of them should be Annexin V staining, see below) and include at least one viability assay (preferably clonogenic capacity) to describe a corresponding phenotype.

One of the events most commonly associated with apoptosis is the exposure of phosphatidylserine (PS) on the outer leaflet of the plasma membrane 182. However, PS externalization might be context-dependent to a certain degree, at least within the complexity of the human cellular network 93,183,184. It remains unclear whether this is also the case in yeast, although the current evidence suggests that PS externalization is a universal feature of yeast cells undergoing apoptosis. PS externalization can be assessed via monitoring PS-binding to Annexin V, which is usually fluorescently labeled for quantitative (e.g., fluorescence reader-based or flow cytrometric analyses) and qualitative (microscopic) evaluation. To this aim, the cell wall needs to be (partially) digested in order to make the externalized PS accessible to Annexin V and permit binding. Usually, the Annexin V assay is performed as a co-staining with a marker for plasma membrane rupture like PI 63,96,104,157. This allows for the discrimination between several subpopulations as they occur in yeast: (i) Annexin V/PI double negative, (ii) Annexin V positive, (iii) PI positive, and (iv) Annexin V/PI double positive cells.

We believe that the second (Annexin V positive) and third (PI positive) subpopulations can be readily interpreted as apoptotic and primary necrotic, respectively, provided that at least one more assay is performed to validate this assumption. For the fourth subpopulation (Annexin V/PI double positive cells), we favor the following interpretation: unlike multicellular animals, a yeast population presumably does not eliminate apoptotic cells via the phagocytic activity of other yeast cells. In the absence of such clearance by scavengers, an apoptotic cell eventually undergoes a metabolic collapse that results in breakdown of the plasma membrane integrity and thus a necrotic phenotype. This phenomenon is termed "secondary necrosis" to discriminate it from "primary necrosis", which describes the phenotype of "cellular necrosis occurring *ab initio*" 185,186. We thus view the above-mentioned fourth subgroup (Annexin V/PI double positive cells) as a late apoptotic population that has undergone secondary necrosis.

Still, these cells might also have undergone secondary necrosis following other cell death subroutines, but at this point there is no evidence for this possibility, which should be evaluated earlier in the cascade of events leading to cellular demise. Importantly, the phenotypical shift from apoptosis to secondary necrosis might reflect defined mo-ecular events and thus be experimentally distinguishable from ACD with necrotic features and primary necrosis also at the functional level 187,188. It could be argued that, in turn, primary necrotic cells might eventually stain for apoptotic markers like Annexin V, thus also yielding AnnexinV/PI double-stained cells. However, necrotic markers do appear without apoptotic characteristics and such primary necrotic populations are stably maintained during long-term physiological conditions like chronological aging. This strongly suggests that primary necrosis can be distinguished from secondary necrosis by the absence or presence of apoptotic markers. Still, no study has yet systematically evaluated this distinction at the cellular level, for instance, via cell sorting analysis. Until such further analysis, this interpretation remains a valid approximation. In any case, we suggest determining the kinetics of the cell death process (see above) to accurately resolve the appearance of these subpopulations. In general, any approaches that facilitate monitoring death scenarios time-dependently represent a helpful improvement, for instance replicative age-associated changes using microfluidic platforms 189,190,191,192,193.

In multicellular animals, clearance of apoptotic cells is a central physiological feature for maintenance of organismal homeostasis. Still, secondary necrosis does occur under certain circumstances 186. *In vitro*, cultured metazoan cells that are left to finalize the apoptotic process without interruption (e.g., without interference of phagocytic scavenging) eventually succumb with features of secondary necrosis 186,194,195. *In vivo*, secondary necrosis may occur in multicellular animals, for example, when apoptotic cells are shed into the lumina of hollow organs with low probability to encounter scavengers or when apoptotic cell death occurs at a pace that surpasses the local scavenging capacity 186,196,197. These observations suggest that secondary necrosis following apoptosis is a conserved outcome upon exposure to pro-apoptogenic stimuli if clearance mechanisms are absent or insufficient.

Besides PS externalization, apoptotic cells exhibit chromatin condensation, which can be readily assessed by nuclear staining with dies such as 4',6-diamidino-2-phenylindole (DAPI) followed by microscopic inspection 63,125. Another characteristic that accompanies yeast apoptosis - especially at late steps of the process - is DNA fragmentation. It is often assessed via the "terminal deoxynucleotidyl transferase-mediated dUTP nick end labeling" (TUNEL) test, which allows for the fluorescent labelling of free 3′-hydroxyl ends that can be easily monitored via microscopy analysis and quantified using a fluorescent plate reader or a flow cytometer 63,96,104,157. In many yeast cell death scenarios, TUNEL positivity matches apoptotic markers determined by other assays 96,104,157,198. However, TUNEL staining detects free 3′-hydroxyl ends regardless of the molecular mechanism involved in generating them. In fact, in some conditions, necrosis, DNA repair, or active gene transcription have all been shown to yield TUNEL positivity, at least in human cells 199,200,201,202,203,204. In yeast, the nature and the kinetics of DNA fragmentation detected by the TUNEL test need further investigation, even though previous studies have partly addressed these issues 79,205. In summary, we recommend using the TUNEL test as a method to determine the occurrence of DNA fragmentation associated with yeast apoptosis rather than a technique for quantifying apoptosis on its own. In addition, the TUNEL test may provide an assay to screen for cellular demise in high-throughput assays. In this setting, hits must be confirmed by testing cellular membrane integrity and clonogenic capacity. Furthermore, apoptotic DNA damage may be tested using the so-called "comet assay", or single cell gel electrophoresis, whereby physiologic DNA strand breaks are distinguished from apoptotic DNA dissolution in individual cells (the latter forms a distinct cluster of fragmented DNA at the ‘tail’ of the comet) 206. In addition, the flow cytometric detection of a subpopulation with hypoploid DNA content (sub-G_0_/G_1_) has been previously employed as an alternative to assess apoptotic DNA degradation 207. However, such results should be interpreted carefully, since apparent hypoploidy may also reflect an artefact from the debris associated with necrotic cells, unless discarded by cell sorting analyses 208.

Apoptotic cell death often follows mitochondrial outer membrane permeabilization (MOMP), which culminates with the release of pro-apoptotic proteins from the intermembrane space and irreversible loss of mitochondrial transmembrane potential (Δ*ψ*_m_) 96,159,160,162,163,164,209,210. A detailed analysis of these mitochondrial subevents requires precise kinetic determinations. For instance, in acetic-acid induced RCD, pro-apoptotic cytochrome *c* release, which depends on the ADP/ATP carrier 211, occurs before mitochondrial integrity is lost 212. All of these biochemical features might be evaluated to determine an apoptotic phenotype, though it should be kept in mind that mitochondria have also been associated with at least one other RCD subroutine (regulated necrosis) 149. Thus, we recommend the involvement of mitochondria in apoptosis to be validated by at least two specific methods (one of them should be assessing PS externalization) and at least one viability assay (preferentially clonogenic capacity).

A large number of apoptotic regulators and executors have been identified in yeast so far 62. This enables testing whether RCD occurring upon a given stimulus is at least partly dependent on one of these factors based on genetic manipulations, pending confirmatory experiments with morphological and biochemical assays. We advise to interpret results from genetic disruption or inhibition studies with caution, as it is difficult to estimate whether other or how many signaling cascades have been affected by a manipulation *a priori* specific. Indeed, many yeast cell death regulators, e.g., cytochrome *c*, apoptosis-inducing factor (Aif1), endonuclease G (Nuc1) and the yeast metacaspase (Yca1), exert both lethal and vital functions 62,69,96,159,160,213,214,215,216. Importantly, the molecular network underlying apoptosis regulation in yeast is starting to be uncovered and additional regulators and subroutines that are yet unknown are expected to emerge. Thus, if a given cell death phenotype is not dependent on any of the known apoptotic regulators this does not exclude apoptosis as a possible cell death modality.

For exploring a putative apoptotic mechanism in a given cell death scenario, the deletion strains of known apoptotic regulators should be harnessed, since distinct apoptotic subroutines exist that rely on different factors that may act independently from each other to orchestrate cellular demise. For instance, the yeast metacaspase Yca1 is involved in many apoptotic RCD and PCD settings 62,69,75. Thus, cell death inhibition in *yca1* knockout strains may point towards an apoptotic mechanism. However, under certain conditions, apoptosis is not executed via Yca1, but instead relies on other factors, including Aif1, Nuc1, the human cyclophilin D ortholog Cpr3, the BH3-only protein Ybh3 or ceramides 96,160,217,218,219,220,221,222,223. Importantly, while yeast harbors a single metacaspase-encoding gene (*YCA1*), it is possible that other proteases might functionally substitute for metacaspases 224,225,226. Thus, in cases where Yca1 is not involved in cell death regulation, we favor the expression "Yca1-independent" instead of "metacaspase-" or "caspase-independent" cell death. For cell death stimuli that are dependent on Yca1, we consider that the terms "Yca1-", "metacaspase-", and "caspase-dependent" are all appropriate. In fact, though much controversy has accompanied the denomination of metacaspases as true homologs of caspases, recent advances strongly indicate that this is the case 71. Indeed, caspases and metacaspases seem to be evolutionary distinct variants with a functional commonality that do fulfill the criteria of homology, since they both share (i) a common cellular program (RCD) and (ii) common or at least overlapping substrates 70,227,228.

In human cells, extrinsic apoptosis defines a caspase-dependent cell death subroutine that is induced by extracellular lethal ligands. These ligands are sensed and transmitted either via specific transmembrane death receptors or through so-called ‘dependence receptors'. Dependence receptors can trigger two opposite signaling pathways: in the presence of ligand, they elicit signals involved in cell survival, migration and differentiation, but in the absence of ligand, they promote apoptotic RCD. Thus, dependence receptors only exert lethal functions when the concentration of their specific ligands falls below a critical threshold level 229. While in yeast no such dedicated receptors are known, cases of metacaspase-dependent apoptosis induction by molecules that may operate from the extracellular microenvironment have been described. For instance, toxins secreted by virus-infected killer strains and a number of drugs have been shown to trigger apoptosis executed by Yca1 82,230,231,232,233. Yet, it remains unknown whether these factors act on intracellular targets, or whether they may also bind to plasma membrane-localized receptors. Given the complexity and interactivity of a yeast population, it is conceivable that a yet-to-be-determined extrinsic apoptotic pathway may co-regulate cell death within a yeast community 79,234. However, and meeting the definition of extrinsic apoptosis put forward by the NCCD, we suggest not to use this term until dedicated death receptors or dependence receptors are discovered. Similarly, another specific type of apoptosis in human cells, anoikis, which defines a form of intrinsic apoptosis restricted to adherent cells that detach from the matrix 235, is theoretically possible in yeast. Indeed, adhesion mediated by cell-wall-bound adhesins is crucial for colony and biofilm formation as well as for host-pathogen interactions 236,237,238. While it remains conceivable that normally adherent yeast cells, which detach in a specific scenario where adhesion is important, might undergo a form of anoikis, this form of RCD has not (yet) been described in yeast.

## REGULATED NECROSIS

In dying yeast, necrotic characteristics may appear in the frame of a primary or secondary necrotic process. While secondary necrosis is probably a consequence of apoptosis in most if not all cases (see above), a primary necrotic phenotype (which occurs without any preceding apoptotic traits) may result from two cell death modalities: ACD or RCD. Indeed, yeast primary necrosis can not only be the outcome of severe insults (accidental necrosis), but also develop as an event orchestrated by a genetically controlled machinery (regulated necrosis) 103. In both cases, cell death is characterized by a set of distinct morphological and biochemical features that defines it as necrotic.

Necrosis first leads to a gain in cell volume and organelle swelling (oncosis), which may be observed, for instance, using fluorescent microscopy of GFP-fused proteins that mark organellar membranes 149. Eventually, necrotic cells also show the complete breakdown and disintegration of subcellular structures, which can be assessed using electron microscopy 157. Similarly, the rupture of the plasma membrane that accompanies necrosis can easily be assayed via electron microscopy or fluorochromes like PI that only enter cells with a disintegrated cell membrane, but are excluded by healthy or early apoptotic cells 103,157. In yeast, the release of intracellular material has not yet been systematically employed as an assay to characterize necrotic cell death. However, the nucleo-cytosolic translocation of Nhp6A may be used to assess necrosis in yeast 104,157,165,239. Nhp6A is the yeast homolog of the mammalian protein high mobility group box 1 (HMGB1), whose release accompanies immunogenic cell death mammalian cells 144,240. We suggest assessing at least two of these markers in order to define *bona fide* primary necrosis in yeast. In addition, viability should be measured with at least one assay (preferably by assessing clonogenic capacity) to corroborate cellular demise. Finally, we strongly recommend to exclude the presence of apoptotic death indicators, and most importantly to differentiate the observed phenotype from secondary necrosis.

As in higher eukaryotic cells, in yeast, ACD may be triggered upon the challenge to extremely detrimental conditions. Thus, agents like hydrogen peroxide, acetic acid, amphotericin B, or several metals that are pro-apoptotic at low doses may induce necrosis at high concentrations 125,218,241,242. We assume that necrosis is the consequence of radical cellular damage in most of these cases, and hence a *bona fide* instance of ACD. This is in line with the concept that not only the type but also the intensity of a given perturbation determines the form of death 91,243.

As mentioned above, yeast can undergo regulated necrosis, reminiscent of the RCD instances detected in human cells 244. Indeed, genetic and chemical manipulations demonstrate that yeast necrosis can be inhibited, at least in some settings, indicating that it results from the activation of a molecular mechanism. In order to differentiate regulated from accidental necrosis, it is necessary to test whether a pharmacological or genetic intervention is capable of inhibiting necrosis in the scenario that is being studied. Known necrosis-modulatory approaches include the exogenous administration of the naturally occurring polyamine spermidine, which can specifically reduce primary necrotic cell death in the context of chronological aging 157. A similar outcome can be obtained by genetic modulation of polyamine biosynthesis 157. In addition, the proteolytically inactive propeptide of the vacuolar endoprotease Pep4, the homolog of human cathepsin D, has been shown to mediate antinecrotic effects. Accordingly, prolonged overexpression of Pep4 (or its propeptide) can extend chronological lifespan via specific inhibition of necrosis 104,245. Intriguingly, the antinecrotic function of Pep4 depends on polyamine biosynthesis 104. In fact, further vacuolar factors as well as other organelles, e.g., peroxisomes, might be connected to regulated necrosis, but this requires further investigation 246,247,248,249,250.

Under certain circumstances, regulated necrosis in mammalian cells may be mechanistically linked to primary Δψ_m_ dissipation 251,252, and such a mitochondrial permeability transition (MPT)-driven necrosis is connected to a series of pathological conditions 253. In yeast, necrotic cell death also seems to depend on mitochondria in several settings 149,165,221. In addition, recent reports show that necrotic cell death upon a lipotoxic insult requires a functional Rim101 signaling cascade that involves the calpain-like protease Rim13/Cpl1 for lethal execution 254,255. To interrogate a possible case of regulated necrosis, it is thus advisable to evaluate a possible mitochondrial involvement. For that purpose, it would be indicated to examine whether necrosis is diminished upon abrogation of mitochondrial function, e.g., in a ρ^0^ strain (which lacks mitochondrial DNA). However, as previously mentioned, mitochondria are the main executors of apoptotic cell death. Thus, mitochondrial dependence cannot be used as a sole determinant to characterize regulated necrosis and must be accompanied by a set of other assays that demonstrate the primary necrotic nature of cell death. Of note, several known mammalian mediators of regulated necrosis have homologs in yeast, including cathepsins, cyclophilin D, calpains, Hsp90, or protein kinase A, among others 244, but only a few of them have been examined in this context 103,256. It will be interesting to see whether these factors possess a conserved necrotic function in yeast, which would expand the possibilities to determine *bona fide* regulated necrosis. Similarly, it remains to be seen whether known inhibitors of regulated necrosis in mammals also interfere with some cell death scenarios in yeast as well 257.

A number of questions remain to be answered with regard to the actual existence of a necrotic RCD subroutine in yeast. In mammals, regulated necrosis plays a number of key roles, most prominently due to its immunogenic nature, for instance upon pathogen infection 244. Such interaction with the immune system, however, is a feature of complex multicellular organisms. Nonetheless, several physiological scenarios in which regulated necrosis seems to be instrumental for yeast, provide a teleological explanation for its existence in a unicellular organism. During chronological aging, for instance, yeast cells die exhibiting markers of both early/late apoptosis and primary necrosis 61,75,157. Interestingly, the fraction of cells dying by primary necrosis actually represents the majority of the dying population that is reduced upon a cytoprotective intervention, at least via polyamine-mediated lifespan extension 104,157. Another example is the necrotic death of the meiotic mother cell during the terminal stages of gametogenesis (sporulation) 149. In this setting, necrosis occurs after the spores have reached the final phases of development, suggesting a controlled coordination that allows for gamete differentiation prior to the elimination of the mother cell. This might well constitute an instance of necrotic PCD, reinforcing the notion that yeast populations must be seen as a multicellular community of genetically identical cells that responds to selective pressures by ensuring the long-term survival of at least one clonal individual. Therefore, it is conceivable that regulated necrosis might participate in cell-to-cell communication via the inevitable release of intracellular contents, as this is the case in higher eukaryotes 244. Such hypothetical necrosis-related quorum-sensing molecules, however, are yet to be identified in yeast.

In human cells, different types of regulated necrosis have been defined, with MPT-driven regulated necrosis and necroptosis among the most extensively studied forms 258,259. In yeast, mechanistic insights into the control of necrosis are still very limited at this point. Thus, we strongly discourage the use of neologisms to avoid confusion. Instead, we propose to employ the term "regulated necrosis" to describe any genetically controlled form of necrosis in yeast (or "programmed necrosis" if it is a form of PCD). Further research into the molecular activators, transducers and executioners of regulated necrosis in yeast will reveal whether potentially different subroutines of the process exist.

## OTHER RCD TYPES

In mammalian cells, a series of other RCD modalities have been defined. Macroautophagy (hereafter referred to as autophagy) is a conserved catabolic process that orchestrates the digestion of intracellular material (e.g., protein aggregates, organelles) in the vacuole. During autophagy, double-membraned vesicles (so-called autophagosomes) form and engulf cytoplasmic components, followed by the fusion of autophagosomes with the vacuole, where the cargo is degraded and the resulting macromolecules are released into the cytoplasm for reuse 16,260. Thus, autophagy is predominantly a cell survival mechanism (see below). Historically, though, "autophagic cell death" (ATCD) was one of the three distinct cell death manifestations (besides apoptosis and necrosis) that were described for human cells based on morphological criteria 261. Although this original description did not indicate any functional connection, it became a widespread belief that ATCD would point to cell death as a mechanistic outcome of autophagy. The term ATCD has indeed been extensively misused to describe cell death instances that occur in the presence of autophagic markers, instead of testing an actual dependency on the process and/or its molecular machinery, i.e., assessing the retardation of cell death via pharmacological or genetic inhibition of autophagy 260. In fact, the NCCD has recently agreed to identify such forms of cell death as "autophagy-dependent cell death" (ADCD) 95. ADCD can in principle describe (i) cell death dependent on the autophagic machinery (in its whole, or parts thereof) and (ii) cell death dependent on actual autophagic degradation. Indeed, (i) components of the autophagic machinery have been etiologically implicated in specific settings of RCD in *Drosophila melanogaster* and human cells 243,262,263,264,265,266. In these contexts, the molecular apparatus for autophagy contributes to cellular demise. To our knowledge, however, there is no study in which cell death has been directly linked to (ii) a functional autophagic flux. Thus, we surmise that most cases of ADCD rather depend on components of the autophagic machinery than on autophagic responses. In fact, the molecular machinery of ADCD and adaptive autophagy partially differ (at least in *D. melanogaster*) 267,268.

In yeast, the term ATCD has been used to describe cellular demise occurring under specific external stress conditions like zinc-induced cell death 269, heterologous expression of human α-synuclein 174 or human p53 270 as well as internal deficiencies like defects in inorganic pyrophosphatases 271. Following the recent proposition by the NCCD, we favor the use of the term ADCD (instead of ATCD) in yeast, as well. Again, ADCD should be used to describe cell death only when autophagy (or at least two proteins from the autophagic machinery, see below) has been experimentally given an etiological implication in the process. As a note of caution, it is important to underscore that the term ADCD should be avoided if the autophagic machinery (or components thereof) is activated parallel to (rather than triggering) RCD or if it promotes other RCD subroutines 95. In fact, in most known cases from yeast to human, autophagy acts as a cytoprotective response to detrimental stress conditions, in which it disposes damaged cellular material 37,272,273. Accordingly, cell death is rather accelerated than repressed upon inhibition of autophagy in both human cells and yeast 274,275,276. In fact, and despite the evidence for autophagy activation in the course of cell death (see above), the very existence of ADCD as an actual cell death type has been questioned 277,278. In any case, cell death may often be preceded or accompanied by autophagy markers, probably mirroring the final effort of dying cells to counteract a lethal stress. Thus, in most cases, cells showing biomarkers of autophagy might be dying with, and not by, autophagy. We thus consider that the use of the term ADCD should be used with utmost care, taking into account the aforementioned NCCD recommendations 95.

A number of microscopic, biochemical and enzymatic assays are available and established 175,279,280 to determine autophagic flux, i.e., the progression through the pathway and thus its degradation activity 175,176. One of the most common methods to measure autophagic flux in yeast is to evaluate the vacuolar processing (or GFP liberation) of N-terminally GFP-tagged Atg8, a central modulator of autophagosome formation, and its delivery to the vacuole, via fluorescence or immunoblot analysis 166,167,168,169,170. Other widely used assays include assessing the autophagy-dependent activity of a modified version of the vacuolar alkaline phosphatase Pho8 via a specific enzymatic assay 118,171 or monitoring the pH-change of cellular compartments upon delivery of pH-sensitive fluorescent proteins to the vacuole (such as Rosella) 173. However, such quantitative assessments - while necessary - are not sufficient to characterize ADCD: for that purpose, a functional dependency on the autophagic machinery (or components thereof) must be concluded, as mentioned above. Thus, all cases of cell death that are accompanied by autophagic markers, but cannot be suppressed or retarded by inhibiting the (at least parts of) the molecular apparatus of autophagy should not be considered as ADCD.

The causative implication of autophagy in cell death may be explored by deletion of autophagy-related (ATG) genes, which are the key orchestrators of the process 175. However, ATGs may have autophagy-unrelated functions as well 281. Thus, akin to the recommendations for higher eukaryotes 260, we suggest testing at least two (and better more) distinct ATG deletions to assess dependency on the autophagic machinery. Inhibitory components of the autophagic apparatus can also be targeted, e.g., by constitutively activating the TOR complex 1 or the RAS/PKA signaling pathway, resulting in autophagy suppression 178,179,180,181. As mentioned above, dependence of cell death on the molecular machinery of autophagy (in its whole, or parts thereof) does not imply cell death to be dependent on autophagic degradation. To evaluate if the autophagic response is implicated in the lethal execution, one may take advantage of chemical inhibition 175,177. Vacuolar proteolysis can be blocked through direct inhibition of proteases either genetically (e.g*.*, by deleting *PEP4* or *PRB1*) or pharmacologically (e.g*.*, by addition of pepstatin A, E-64D, leupeptin alone or in combination) as well as by neutralizing the vacuolar pH (e.g*.*, by means of chloroquine) 175,176. In yeast, chemical inhibition of autophagosome formation (as it is commonly applied in mammals using specific suppressors of phosphatidylinositol 3-kinase) is not typically employed, since substantially higher concentrations of these drugs are often needed 262,265. In fact, genetic approaches are generally favored in the ADCD field due to insufficient specificity of most pharmacological autophagy inhibitors 260.

The expression "mitotic catastrophe" (MC) was first employed to illustrate the lethal phenotype of a temperature-sensitive fission yeast mutant strain that enters mitosis prematurely without effectively completing it 11. The term MC has since been most frequently used to define cell death that occurs upon aberrant mitosis 94, which is frequently accompanied by gross nuclear alterations. In yeast, as in mammals, it may result from genome instability, microtubule destabilization, DNA damage, or alterations in cell cycle checkpoints 282,283,284,285. Intriguingly, yeast RCD has been connected to most of these features 63,286,287,288. It will be interesting to follow whether known MC scenarios culminate in specific RCD subroutines.

In mammalian cells, death following mitotic aberrations can, indeed, be either apoptotic or necrotic 289. Since mitotic defects may contribute to malignant transformation in the mammalian system, MC can be viewed as an oncosuppressive mechanism that operates via cell death or senescence 94,289. In fact, suppression of MC provokes tumorigenesis and cancer progression in mammals 290. By analogy, MC in yeast might be a mechanism to eliminate mitosis-incompetent and thus unfit cells from the population. Adhering to the recommendations by the NCCD 94, we thus propose to use the term MC as an independent molecular avenue that precedes RCD, but does not constitute a *bona fide* cell death executioner mechanism by itself 290.

A series of other cell death subroutines have been defined in human cells that, however, are restricted to specific cell types and thus do not apply to yeast.

## RCD IN OTHER YEASTS AND FILAMENTOUS FUNGI

As previously mentioned, yeast cell death has been most extensively studied in *S. cerevisiae*. However, other yeast species have been shown to share similar cell death characteristics and also bear a set of comparable cell death subroutines. Thus, we propose to extend the above-described recommendations formulated above to all yeast species.

*Schizosaccharomyces pombe* (fission yeast) has been shown to express an RCD machinery that responds to various stimuli. These include physiological triggers such as aging, defects like the abnormal metabolism of intracellular lipids 291,292,293,294, and a number of insults, including ER stress 295, inositol starvation 292,293 or the heterologous expression of several metazoan apoptotic effectors, e.g., BAX and BAK 296. All of these stimuli converge on the activation of apoptosis. Of note, according to our definitions, neither regulated necrosis nor ADCD have been demonstrated in *S. pombe* (yet). Among the described *S. pombe* apoptosis executors are the chaperone Cnx1 (calnexin) and the metacaspase Pca1 295,297. Pca1 is involved in the apoptotic response to inositol starvation 295,297 and lipid-induced, non-apoptotic cell death in minimal medium. Conversely, Pca1 does not seem to play any role during apoptosis induced by ER stress 295, valproic acid treatment 298, or lipotoxic stress in minimal medium 292. *S. pombe* apoptosis is expected to involve additional players, as there is evidence for the presence of different factors in fission yeast that are homologous to effectors of *S. cerevisiae* apoptosis, including the protease Nma111 299, Aif1 300 or endonuclease G 301.

The major opportunistic human pathogen *Candida albicans*, which has become a molecular genetics model to study pathogenicity, virulence and fungal development 302,303, can also undergo apoptosis following the exposure to a plethora of different agents 242,304,305,306. To date, no RCD subroutines other than apoptosis have been described. Interestingly, apoptosis can occur in both the blastospore and the hyphal form of this organism 305 as well as in *Candida* biofilms, which are highly tolerant to standard antimycotics and hence difficult to eradicate. Exploiting the apoptosis machinery in cells constituting biofilms may pave the way to their effective eradication, and hence limit the incidence of indwelling device-associated infections (IDAIs) 307,308,309. *C. albicans* also harbors a gene encoding a metacaspase (*CaMCA1*) 310, which mediates apoptosis, for instance, upon treatment with farnesol 311, caspofungin 312, and micafungin 232 or upon interaction with murine macrophages 311,313. Conversely, CaMca1 is not involved in other apoptotic settings like exposure to the plant defensin RsAFP2 314. The Ras-cAMP-PKA signaling pathway 315 and the bZip transcription factor Cap1 316,317 have also been implicated in distinct apoptotic scenarios. Finally, other closely related *Candida* species, e.g., *Candida glabrata* 318, *Candida krusei* 319, *Candida dubliniensis* 320, *Candida tropicalis* 321, or *Candida parapsilosis* 232,322, have been reported to exhibit apoptotic markers upon lethal challenge.

*Cryptococcus neoformans*, an important pathogen of immunocompromised and immunocompetent patients, also undergoes apoptosis 323,324, with apoptosis-inducing factor and two metacaspases independently orchestrating this lethal subroutine 324. At least one other *Cryptococcus* genus member, *Cryptococcus laurentii*, has also been shown to respond to some stimuli with apoptotic RCD 325. Furthermore, a number of other yeast species, e.g*.*, *Kluyveromyces lactis* 326,327, *Pichia pastoris* 328, *Rhodotorula glutinis *329, or* Zygosaccharomyces bailii* 330,331, may develop signs of apoptosis under certain conditions. We surmise that similar lethal programs are to be discovered in other yeast species. In fact, such discoveries and further characterization of both identified and yet uncovered RCD programs are expected to follow in the near future, given that antifungal therapeutics for medical and industrial purposes may increasingly rely on targeting the yeast RCD machinery 332,333. We thus suggest adopting the recommendations formulated above for the description of cell death in all types of yeasts.

It should be noted that a growing body of work is addressing RCD in multicellular fungi. A major human pathogenic fungus that causes life-threatening disease is *Aspergillus fumigatus*, which has also been demonstrated to undergo apoptosis under certain conditions 334,335. The genome of *A. fumigatus* codes for two metacaspases (CasA and CasB), whose relative contribution to cell death seems to depend on the scenario 335,336,337. In fact, other fungal proteases might also exert metacaspase activities that are relevant for cell viability and/or survival 337. *Aspergillus nidulans* is another member of the *Aspergillus spp*. that has been demonstrated to undergo RCD 338,339. The genome of *A. nidulans* appears to code for an apoptotic machinery with relevant players like apoptosis-inducing factor and two putative metacaspases 339,340. Another filamentous fungus, *Podospora anserina*, is used as an aging model that incorporates crucial apoptotic factors, including two metacaspases (PaMCA1 and PaMCA2) and at least five Aif members, of which only mitochondrial (but not cytosolic) isoforms seem to be relevant for aging-driven RCD 341,342. A role for the *P. anserina* cyclophilin D ortholog in RCD 343,344,345 as well as for autophagy in aging and lifespan control of *P. anserina* 346,347 have been reported. Further instances of fungal RCD 306,348 have been documented in *Paracoccidioides brasiliensis *349, *Colletotrichum gloeosporioides *350, *Fusarium oxysporum *351, *Fusarium graminearum *352, *Mucor racemosus *353, *Botrytis cinerea *354, *Penicillium expansum *355, *Rhizopus oryzae *356, *Scedosporium prolificans *357 and *Neurospora crassa *358*. *As multicellular organisms, filamentous fungi have developed programs that are reminiscent of organismal RCD. For instance, several putative homologs of factors relevant for animal apoptotic control that are not found in unicellular yeast are present in the genomes of filamentous species 359. Thus, multicellular fungi may have complex traits not present in yeasts that may add to the criteria and definitions presented herein.

## CONCLUDING REMARKS

The impact of yeast (and other fungi, including filamentous species) on our lives at multiple socioeconomic, scientific and medical levels emphasizes the importance of decoding the mechanisms that determine its survival and control its demise. Therefore, the molecular comprehension and potential manipulation of yeast cell death hold major promise for biotechnological and biomedical applications. We anticipate that numerous fields might benefit from the possibility to modulate yeast cell death. For instance, the productivity of yeast during large-scale processes in the pharmaceutical and industrial arenas largely depends on its viability and ultimately on its tolerance to stress and its demise in stationary cultures. Also, novel pharmacological approaches that specifically target the RCD machinery of yeast pathogens may bypass the ever-increasing resistance to classical antimycotics, which is an emerging public health problem. Other medical manipulations of yeast RCD are also conceivable, e.g., strategies to intervene on pathogenic deviations of the mycobiome. Finally, yeast will continue to help the community in deciphering eukaryotic cell death pathways as it serves as an important model for human disease. Given its power to study the relationship between genotype and phenotype, we expect to gain further insights from yeast to identify actionable targets that may be subjected to pharmacological (drug discovery) or genetical manipulation.

For all these reasons, it is now imperative to set the standards for defining and studying cell death in yeast. That said, we want to emphasize that the present set of recommendations should be taken - as any scientific overview - as a snapshot of the current knowledge, rather than as a definitive compilation. Indeed, as research continues, we surmise that the present guidelines will have to be extended and revised. For instance, other nuanced changes to - or even novel types of - RCD may emerge from continued efforts to characterize the multicellular character of yeast populations, including but not restricted to uncovering intercellular communication, interaction between populations or cellular differentiation within colonies and biofilms. Still, neologisms should be introduced with care and only when the characterization of a lethal process that bears new functional and biochemical aspects requires it. Otherwise, new expressions should be avoided to limit confusion.

Another crucial point is to acknowledge the inherent complexity and dynamic nature of RCD in general and its different subroutines in particular. In fact, it is the crosstalk between pro-life and pro-death signals that determines cellular fate, and the activation of pro-survival pathways (such as autophagy) may often accompany lethal signals. Also, stress conditions may activate different RCD subroutines that can be interconnected or may occur independently, sequentially, or in parallel. Indeed, the inhibition of one specific RCD modality might trigger backup mechanisms that still ensure cell death execution. It is thus important to keep these points in mind when classifying a lethal phenotype.

Altogether, the present guidelines attempt to unify the nomenclature and definition of yeast cell death modalities and - in our opinion - will help other fields of unicellular research (e.g., bacteriology, parasitology, etc.) to establish their set of recommendations using the present one as a basis. We are convinced that some degree of linguistic and experimental standardization is necessary for facilitating communication among researchers, especially at a point where the existence of yeast RCD is scientifically accepted and its socioeconomical impact is ever growing.
